# Patient experiences and perceived efficacy of a newly implemented hospital-based withdrawal management unit in Vancouver, Canada: findings from the Road to Recovery evaluation

**DOI:** 10.1186/s13722-026-00671-5

**Published:** 2026-04-30

**Authors:** Sidney Roberge, Seonaid Nolan, Cheyenne Johnson, Piper Dickhout, Ava Abramowich, Travis De Wolfe, Renee Janssen, Erika Mundel, Andrea Ryan, Ian Haynes, Laurel Lemchuk-Favel, Harmony Johnson, Brittany Dennis

**Affiliations:** 1 Faculty of Medicine, University of British Columbia, Vancouver, Canada; 2 British Columbia Centre on Substance Use, Vancouver, Canada; 3 Department of Medicine, University of British Columbia, Vancouver, Canada; 4 Division of Addiction, Providence Health Care, Vancouver, Canada; 5 Indigenous Wellness and Reconciliation, Providence Health Care, Vancouver, Canada; 6 Division of Social Medicine, Department of Medicine, University of British Columbia, Vancouver, BC Canada

**Keywords:** Addiction medicine, Substance use, Health service evaluation, Withdrawal management, Quality improvement, Patient experience, Integrated care

## Abstract

**Background:**

The Road to Recovery (R2R) initiative represents an innovative approach to substance use care in British Columbia, Canada, that is designed to provide timely, comprehensive, and culturally safe services for individuals with a substance use disorder (SUD). This survey sought to understand patient experiences with this new care model.

**Methods:**

Adults (≥ 18 years) with a SUD who accessed R2R’s new hospital-based withdrawal management unit in Vancouver, Canada between May 2024 and April 2025 were invited to participate in a cross-sectional survey that captured their individual experiences. Descriptive statistics were used to summarize responses.

**Results:**

87 participants completed the survey. The mean age was 41 years (Standard Deviation [SD] = 15); 57% (*n* = 50) identified as male, and 49% (*n* = 43) as Indigenous. The majority (*n =* 58, 67%) of respondents reported access to withdrawal management services in *≤* 48 h. A total of 91% (*n* = 79) of participants felt the quality of care was ‘good’ or ‘excellent’, 90% (*n* = 78) reported positive interactions with staff, 88% (*n* = 68) of respondents felt their withdrawal symptoms were adequately managed, 91% (*n* = 71) believed the program helped them achieve their treatment goals. Among participants visited by Indigenous Wellness Liaisons (IWLs) *(n* = 56), 77% (*n =* 43) completely trusted IWLs, and 52% (*n =* 17) reported that their cultural and spiritual needs were completely met by IWLs. Peer support was valued by 85% of respondents for providing empathy and motivation. One-third (*n =* 29, 33%) of participants contemplated leaving during their admission, most commonly because of the absence of engaging activities (*n* = 12, 41%,). Overall, 95% said they would recommend the program to others.

**Conclusions:**

Findings highlight that the R2R model delivers timely, high-quality, and culturally safe withdrawal management care. Patients emphasized the value of respectful staff, effective medical management, and integration of Indigenous and peer supports. Enhancing structured programming and expanding access to psychosocial and cultural services may further improve engagement and reduce early discharge. Embedding trauma-informed, interdisciplinary, and culturally grounded approaches within hospital-based withdrawal management may improve patient experience and retention, informing future models of addiction care in Canada.

**Supplementary Information:**

The online version contains supplementary material available at 10.1186/s13722-026-00671-5.

## Introduction

Canada is facing a public health emergency due to the rising number of toxic-drug deaths. Since 2014, the rate of unintentional drug poisoning deaths nearly doubled from 6.4 to 11.5 deaths per 100,000 [1]. Between January 2016 and September 2024, Canada recorded 50,928 drug toxicity deaths, of which 75% involved fentanyl and 68% involved unregulated stimulants [2]. The crisis is being driven by potent synthetic opioids like fentanyl and further exacerbated by the emergence of benzodiazepines and other adulterants (e.g. medetomidine) in the unregulated drug supply, both of which have contributed to atypical withdrawal symptoms with serious adverse consequences, including seizures [3, 4].

As the drug supply continues to evolve, traditional approaches to managing withdrawal are becoming less effective [5]. As a result, conventional outpatient services and community-based withdrawal management programs are challenged to meet the complex medical needs of the increasing number of patients experiencing severe complicated withdrawal, and those with significant comorbid medical (i.e., cirrhosis) and mental health conditions [5–7]. Prior research suggests that integrating withdrawal management services within an acute-care setting has several advantages. Firstly, this setting ensures individuals at highest risk for severe, complicated withdrawal (e.g., seizures, death) can be managed in a clinical environment by health care staff with the appropriate expertise [8]. Furthermore, managing one’s complex withdrawal in the inpatient setting affords the opportunity for more aggressive approaches to the initiation and titration of evidence-based addiction medications (e.g., more rapid titration of opioid agonist medications, more frequent administration of adjuvant medications, etc.) [9]. In addition, mainstream addiction services in Canada and the U.S. remain rooted in Eurocentric models that overlook the cultural, historical, and social realities of Indigenous peoples, contributing to ongoing inequities in care and outcomes. Research consistently calls for Indigenous-led and community-driven approaches to address the colonial roots of these disparities and to ensure the model of care being delivered is both trauma-informed and culturally safe [10–12]. These challenges highlight the need for withdrawal management models that can provide intensive medical monitoring, interdisciplinary care, and culturally safe services for diverse patient populations to improve engagement with addiction treatment services, including withdrawal management.

Evaluating patient experiences is essential to ensure new models of care consider and respond to factors contributing to the historically high rates of patient-initiated discharge (PID), including environments that patients find unsafe or unwelcoming [13]. Incorporating patient feedback is a key approach to this challenge, addressing a long-standing gap in addiction care where services have often been shaped without meaningful engagement from those they serve and their caregivers/loved ones [14, 15]. To date, the majority of research aimed at improving acute withdrawal management services have focused on retrospectively identifying factors linked to patient-initiated discharge, with no large-scale studies yet evaluating patient experience during program design and implementation [13, 16, 17]. This survey seeks to capture patient experiences accessing substance use care in the Road to Recovery’s hospital-based withdrawal management unit, providing insights that can guide the development of comprehensive, culturally safe care.

## Methods

### Survey summary

Individuals with a substance use disorder (SUD) that were admitted to the R2R’s hospital-based withdrawal management unit at St. Paul’s Hospital (SPH) in Vancouver, Canada between May 2024 and April 2025 were invited to complete a cross-sectional survey that detailed substance use patterns and treatment goals (prior to admission) as well as individual experiences with healthcare staff and clinical service delivery during admission. As a quality improvement initiative, this survey was exempt from Research Ethics Board review and did not require informed consent.

### Setting and participants

**Setting** The Road to Recovery initiative was opened in 2023 as an innovative model of substance use care in the Vancouver Coastal Health region of British Columbia that seeks to: (1) increase treatment capacity through the creation of 121 net new substance use treatment beds (25 of which are new hospital-based withdrawal management beds); and (2) improve operational efficiency within existing acute and community-based substance use services in the region. The health authority in which it operates observes a disproportionately high number of unregulated drug deaths compared to the rest of the province (33.7 versus 30 deaths per 100,000 respectively in 2025), and provides care to individuals in the region triaged at highest risk for severe complicated withdrawal [18]. Admission to the unit is voluntary, and individuals must demonstrate a desire to change their relationship with substances to be eligible for admission, whether that be abstinence, reduced frequency of use, managing withdrawal safely, or assistance in transitioning to ongoing inpatient or community based treatment.

It consists of an interdisciplinary care team comprised of nurses and social workers (with expertise in substance use care), a pharmacist, and peer support specialists (individuals with lived or living experience of substance use). Furthermore, a partnership was created early in the pre-implementation phase with the hospital’s Indigenous Wellness and Reconciliation (IWR) team in order to integrate Indigenous cultural safety into the R2R model from the outset. Indigenous cultural safety requires healthcare professionals and organizations to critically examine their own biases, assumptions, and structures and work to ensure that care – defined as safe by Indigenous patients and families – reduces bias and advances health equity and Indigenous human rights [19]. Accordingly, a range of systemic efforts have been undertaken throughout the R2R model of care to address and advance Indigenous cultural safety. Indigenous Wellness Liaisons (IWLs) are an integral component of the team to ensure Indigenous patients have access to the type of spiritual/cultural supports that they need (e.g., access to Elders, smudging, talking circles, traditional foods). The IWR team maintains a strong relationship with a group of Indigenous Elders whom they work closely with, as Elders and IWLs are distinctly separate positions on the unit. Elders are drawn in to perform specific ceremonies and provide specific support to patients as needed. There is significant cultural diversity among the Elders and IWLs, enabling this team to provide care for a broad group of Indigenous patients, who come from diverse Nations across the country. Additionally, specific Indigenous-focused care the unit provides includes the provision of traditional foods (Pacific salmon, berries, pemmican/jerky, seaweed), traditional herbs, and teas. These supports are offered on the unit with the goal of providing culturally safe care, defined as care that recognizes and addresses the social, history, and political contexts that affect healthcare interactions. It is determined by the experience of the patient, not by the healthcare provider, and requires ongoing self-reflection by providers and organizations [19]. The space itself was also designed with cultural safety in mind, and features photography of native plant life that was traditionally used by Indigenous people which are labelled in English as well as in the languages of the local Nations. Figure S1A features a western red cedar that was used by Indigenous people for spiritual cleansing and ceremony, as well as building long houses, canoes, bedding, and baskets. Figures S1B depicts the common camas which was traditionally used for food and medicine.

There are no restrictions on the allowed length of stay, patients remain on the unit until withdrawal is successfully managed and disposition can be determined. The average length of stay on the unit is 5 days. The unit is funded through a single payer universal healthcare system with clinical services directly funded through British Columbia Ministry of Health. Further funding which supported the initial implementation and evaluation have been provided through philanthropic donation.

#### Participants

Patients were eligible to participate in this survey if they were 18 years or older, were admitted to the R2R withdrawal management unit, and willing to complete the cross-sectional survey in English. Patients who were unable to adequately complete the survey due to unstable medical or psychiatric illness were excluded.

#### Recruitment

Posters advertising the survey were mounted on the unit and participants were able to self-refer for survey inclusion. Eligible participants may also have been approached by a member of the research team to discern interest in completing the survey. Recruitment for the survey was timed as close to discharge as possible to ensure participants had sufficient experience on the unit to inform their responses. Honorarium consisted initially of candy (following completion of the survey) that was subsequently transitioned to a $10-dollar gift card during the last 2 months of recruitment (March and April 2025) to address low survey numbers. Surveys were self-administered, though members of research staff were available to address any participant questions or those in need of support.

### Instrument design and data collection

#### Survey design

The cross-sectional survey was developed with input from the unit’s clinical and operational teams, the organization’s IWR team, peer support specialists, and members of the R2R’s Patient and Family Advisory Committee. Initially intended to measure patient satisfaction and garner feedback for clinical service improvement, the survey was expanded based on stakeholder input to capture more comprehensive data on 5 key domains including: (1) substance use patterns and treatment goals (at admission); (2) interactions with staff and other patients; (3) access and experience with cultural and psychosocial services; (4) satisfaction with the unit’s physical environment; and (5) overall experience with the R2R model of care. The survey includes multiple-choice and 5-point Likert scale questions and an ‘other’ option to allow flexibility to answers provided. An open-ended text box was included at the end of the survey to allow respondents to provide additional feedback not adequately reflected in the survey’s pre-defined categories.

Validated questions adapted from the Hospital Consumer Assessment of Healthcare Providers and Systems (HCAHPS) survey, a standardized and validated tool developed by the Centers for Medicare & Medicaid Services (CMS) to measure patients’ perceptions of their hospital experience, were included in the cross-sectional survey [20]. The HCAHPS survey assesses key domains such as communication with healthcare providers, responsiveness of hospital staff, cleanliness and quietness of the hospital environment, discharge information, and overall hospital rating [20]. Adapted items were incorporated into the survey to assess patient interactions with healthcare workers (e.g., respect, communication, attentiveness), satisfaction with the care environment (e.g., comfort, privacy), understanding of care received (e.g., treatment plans and medications), and preparedness for discharge.

Considering that the IWR team had a key role in the planning and implementation of R2R, they helped to co-develop the survey, oversee its implementation and recruitment strategy, and ensured data governance aligned with Indigenous data sovereignty principles. Questions on identity, cultural supports, cultural safety, traditional healing practices, and interactions with Indigenous providers were developed with the IWR team. Language used to capture Indigenous identities within the survey (First Nations, First Nations and Métis, and Métis) were reviewed and approved under the guidance of the IWR team. Distinguishing First Nations and Métis identifies is critical to reflect the distinct cultural and historical experiences of these Indigenous peoples. First Nations have unique traditions and histories that predate European contact, while Métis peoples have mixed Indigenous and European ancestry and a unique cultural and linguistic identity.

#### Pilot testing and implementation

Survey administration began in May 2024 (9 months following operationalization of the new unit to allow for internal stabilization related to workflows and processes). Responses were anonymous and were securely collected on paper, then inputted into REDCap, a secure, web-based platform hosted by Providence Health Care (PHC) [21].

### Data analysis

Descriptive statistics were used to summarize demographic and survey data. Means and standard deviations were reported for continuous variables, frequencies and percentages for categorical variables. Analyses were conducted in Microsoft Excel [22]. For questions with under 10% missing data, total sample size was used as the denominator; for those with over 10% missing data (marked with ^*^ throughout results), the percentages reflect the proportion of total respondents to those questions. The 10% threshold was selected in accordance with prior studies, which have demonstrated that missing data below this level is unlikely to introduce bias or meaningfully affect the results [23, 24].

## Results

Between May 2024 and April 2025, 87 participants completed the survey (Table 1). The mean age was 41 years (standard deviation [SD]:15), 57% (*n =* 50) self-identified as male, 68%^*^ (*n* = 45) as ethnically white, and 49% (*n* = 43) as Indigenous. Among Indigenous participants, 63% (*n* = 27) identified as First Nations, 12% (*n* = 5) First Nations and Métis, and 12% (*n =* 5) as Métis.

**Table 1:** Demographics, previous withdrawal management access and treatment goals, May 2024 - April 2025 (*n* = 87)

Demographics	Total* n = 87 (%)
**Gender Identity** ^**a**^	
Man	50 (57)
Woman	33 (38)
Non-binary	3 (3)
Transgender	2 (2)
Two-spirit	4 (5)
**Do you identify as Indigenous?** ^**a**^	
Yes	43 (49)
No	40 (46)
Prefer not to answer	2 (2)
** If answered ‘yes’ above**,** what is your Indigenous Identity? (*n*** **= 43)**	
First Nations	25 (76)
First Nations and Métis	8 (24)
Métis	5 (12)
Other^1^	6 (13)
**Ethnicity (*n*** **= 66)**	
White	45 (68)
Middle Eastern	3 (5)
South Asian	2 (3)
Other^2^	16 (24)
**Previous withdrawal management and future treatment goals**	
**Number of times accessing detox in the last 12 months**	
0 times	19 (22)
1 time	22 (25)
2–3 times	34 (39)
4–5 times	5 (6)
> 5 times	7 (8)
**Number of times wanting to go to detox in the last 12 months but unable to do so** ^**b**^	
0 times	33 (38)
1 time	16 (18)
2–3 times	16 (18)
4–5 times	9 (10)
> 5 times	12 (14)
**Goal(s) when accessing care at R2R** ^**c**^	
To stop using all substances	49 (56)
To treat withdrawal symptoms	50 (57)
To reconnect with family and loved ones	35 (40)
To get into a better environment to stop substances	31 (36)
To start on long-term medication	26 (30)
To start an aftercare plan	25 (29)
To understand what resources exist	25 (29)
To work on applications for bed-based treatment	24 (28)
To stop substances so I can be with my children	13 (15)
Other^3^	11 (13)

* signifies over 10% missing data, the percentages reflect the proportion of total respondents to those questions

Imputations for incomplete responses presumed respondents did not check any items for these questions. Percentages reflect a denominator of 87 except in identified cases

a: Missing data: *n* = 2; b: missing data: *n* = 1, c: missing data *n* = 8

1:Other responses included: First Nations and Métis and Inuit, Cree, Assiniboine, unknown; 2:Other responses included: Chinese, other Asian, Latin American, black American, black Caribbean, Persian, Peruvian, European; 3:Other responses included: Quitting specific substances, reducing use, to get back to work/school, to find myself

### Substance use patterns, previous withdrawal management access, and treatment goals

Participants reported a wide range of substance use patterns within the 30 days prior to admission, with alcohol (*n* = 61, 70%), nicotine (*n* = 51, 59%), and fentanyl (*n* = 36, 41%) the most commonly reported substances of use (Figure S2). A total of 78% (*n* = 68) of participants reported accessing withdrawal management at least one time in the 12 months previously, while 62% (*n =* 53) answered that they wanted to access withdrawal management at least once in the previous 12-month period but were unable to (Table 1). Wait times for previous withdrawal management admissions had considerable variability among participants, with 35% (*n =* 31) of respondents reportedly having to wait more than 7 days. When seeking withdrawal management support during this admission, 67% (*n* = 58) of participants reportedly obtained admission to R2R’s withdrawal management unit within 1 to 2 days; 27% (*n =* 24) of participants waited over 2 days.

When asked about treatment goals during participants’ R2R admission, the most common response was “to treat withdrawal symptoms” (*n* = 50, 57%), followed by “to stop using all substances” (*n* = 49, 56%) and “to reconnect with family and loved ones” (*n* = 35, 40%) (Table 1).

### Interpersonal experiences with staff and other R2R patients

#### Staff experiences

When asked about experiences with staff, 90% (*n* = 78) of participants reported having positive experiences, they characterized the staff as friendly (*n* = 72, 92%) and attentive (*n* = 66, 85%) (Table 2). Although the majority of participants had positive experiences, a small number (*n* = 11, 17%) reported having negative experiences with staff due to feeling judged, ignored, or felt staff were inattentive (Table 2).

**Table 2:** Experiences with staff and other patients, May 2024 – April 2025 (*n* = 87)

Reported experiences with R2R staff	Total* n = 87 (%)
**Positive experiences with staff?** ^**a**^	
Yes	78 (90)
No	2 (2)
** If answered ‘yes’ above**,** factors that contributed to positive experiences? (*n*** **= 78)**^b^	
Staff were friendly	72 (92)
Staff were attentive	66 (85)
I felt listened to	64 (82)
Medications were provided on time	63 (81)
I did not feel rushed	57 (73)
Other^1^	8 (10)
**Negative experiences with staff? (*n*** **= 66)**	
Yes	11 (17)
No	55 (83)
** If answered ‘yes’ above**,** factors that contributed to negative experiences? (*n*** **= 11)**^c^	
Felt judged	5 (45)
Staff were inattentive	4 (36)
Felt ignored	3 (27)
Felt like staff did not listen to me	2 (18)
Felt like staff rushed my treatment	2 (18)
Did not provide medications in time	1 (9)
Other^2^	7 (64)
Reported experiences with other R2R patients	
**Positive Experiences with other patients? (*n*** **= 78)**	
Yes	75 (96)
No	3 (4)
** If answered ‘yes’ above**,** factors that contributed to positive experiences? (*n*** **= 75)**^b^	
Other patients respected my privacy	67 (89)
I could relate to other patients	51 (68)
Other patients made me feel safe	46 (61)
Other patients helped my recovery	35 (47)
Other^3^	3 (4)
**Negative experiences with other patient? (*n*** **= 75)**	
Yes	6 (8)
No	69 (92)
** If answered ‘yes’ above**,** factors that contributed to negative experiences? (*n*** **= 5)**	
Other patients were intrusive in my care	2 (40)
Felt triggered when offered substances	2 (40)
Felt unsafe/threatened	1 (20)
Felt judged by other patients	1 (20)
Other^4^	3 (60)

* signifies over 10% missing data, the percentages reflect the proportion of total respondents to those questions

Imputations for incomplete responses presumed respondents did not check any items for these questions. Percentages reflect a denominator of 87 except in identified cases

a: Missing data: *n* = 7; b: missing data: *n* = 2; c: missing data: *n* = 1

Other Responses: 1:Other responses included: empathy and genuine care, feeling comfortable and safe, being generally great people; 2:Other responses included: lack of consistency, insensitivity, not addressing special needs, was not taken seriously; 3:Other responses included: good conversations, made good friends, no gossiping; 4:Other responses include: Triggered by stories or substance use on the unit, was given substances by another patient

**Fig. 1:**
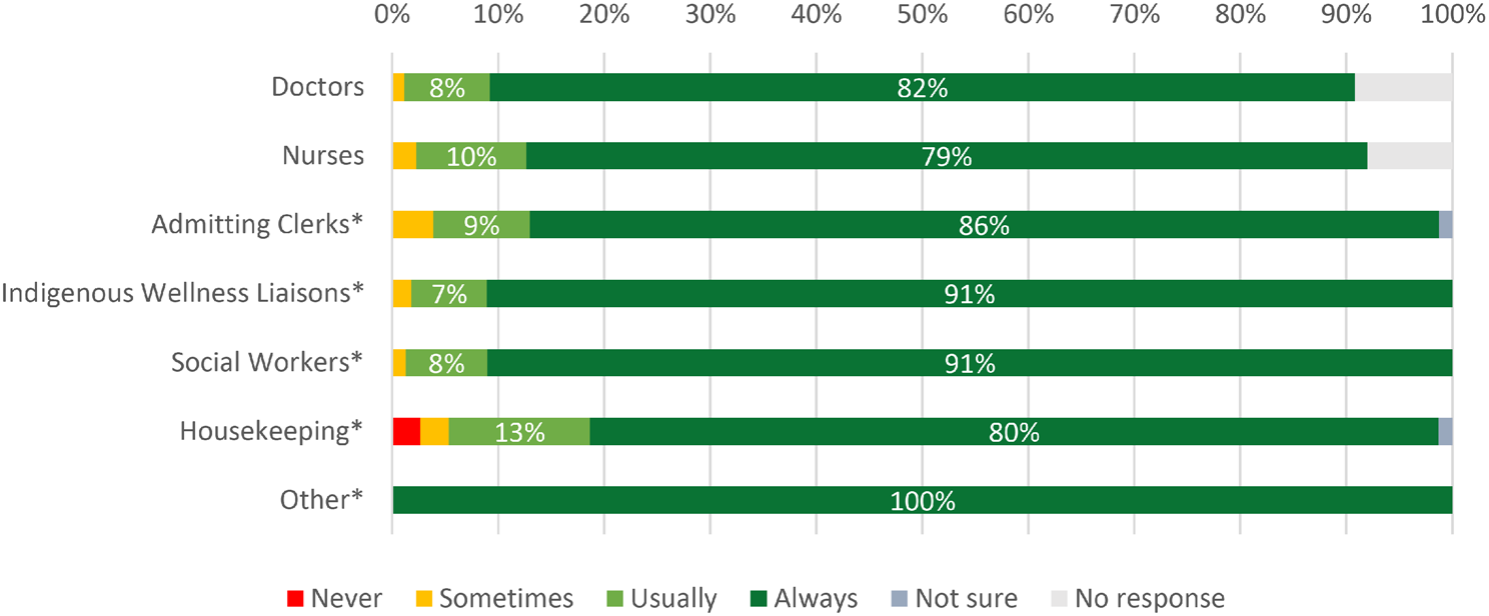
Subjective experiences of respect by the interdisciplinary care team, May 2024 – April 2025 (*n* = 87). Percentages reflect a denominator of 87 except in specified cases. The ‘Admitting Clerks’ category had over 10% of responses missing, percentages reflect a denominator of 77 total respondents. Some categories had differences in denominator based on interaction with this service. The ‘Indigenous Wellness Liaison’ category reflects a total of 56 respondents, the ‘Social Workers’ category reflects a total of 78 respondents, the ‘Housekeeping’ category reflects a total of 78 respondents, while the ‘Other’ category contained 3 responses which included OT and pharmacists. Respondents who indicated they had no interaction with the listed care team member are not reflected in the denominator. Light grey bars indicate no response from participants who make up less than 10% of the total respondents

When asked about being treated with respect by members of the interdisciplinary care team, nearly all respondents indicated feeling they were ‘always’ or ‘usually’ treated with respect by doctors (*n* = 78, 90%), nurses (*n* = 78, 90%), social workers (*n* = 77, 99%*), and admitting clerks (*n* = 73, 95%). Among the 56 respondents who reported having had interactions with the R2R’s Indigenous Wellness Liaisons, 98% (*n* = 56) of respondents felt they were ‘usually’ or ‘always’ treated with respect (Fig. 1).

**Fig. 2:**
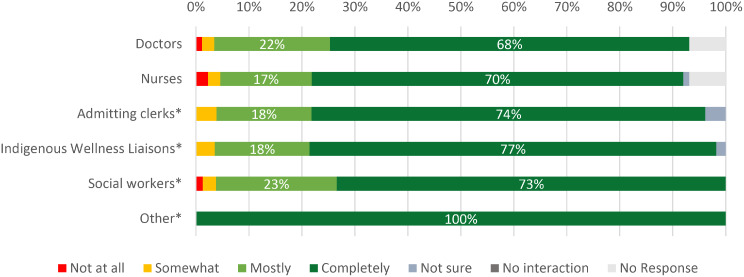
Subjective experiences of trust of the interdisciplinary care team, May 2024 – April 2025 (*n* = 87). Percentages reflect a denominator of 87 except in specified cases. The ‘Admitting Clerks’ category had over 10% of responses missing, percentages reflect a denominator of 78 total respondents. The ‘Indigenous Wellness Liaison’ category reflects a total of 56 respondents, the ‘Social Workers’ category reflects a total of 79 respondents, while the ‘Other’ category contained 3 responses which included OT and pharmacists. Respondents who indicated they had no interaction with the listed care team member are not reflected in the denominator. Light grey bars indicate no response from participants who make up less than 10% of the total respondents

When asked about their level of trust in their providers, nearly all respondents indicated feeling they ‘mostly’ or ‘completely’ trusted the doctors (*n* = 78, 90%), nurses (*n* = 76, 87%), social workers (*n* = 76, 96%*), and admitting clerks (*n* = 72, 92%*). Among the 56 respondents who reported having interactions with the Indigenous Wellness Liaisons, 95% (*n* = 53) of respondents stated they ‘mostly’ or ‘completely’ trusted them (Fig. 2).

Finally, communication with staff during admission and discharge processes was perceived to be effective and culturally safe. During registration, for example, participants were asked if they identify as Indigenous - the majority (*n* = 34, 83%) of respondents who said ‘yes’ reported feeling safe when asked this question, 2% (*n* = 1) felt unsafe, and 10% (*n* = 4) were not asked. Furthermore, when asked about readiness for discharge, 84% (*n* = 73) of participants felt they had a good understanding of the things they were responsible for in managing their health, with only 7% (*n* = 6) of participants feeling that they had a poor understanding.

#### Experiences with other R2R patients

The vast majority of respondents (*n* = 75, 96%*) reported having positive experiences with other patients during their stay at the R2R unit. Among those who endorsed positive experiences, participants noted that other patients respected their privacy (*n* = 67, 89%), that they could relate to others (*n* = 51, 68%), and that co-patients made them feel safe (*n* = 46, 61%). Nearly half (*n* = 35, 47%) of respondents indicated that their peers contributed positively to their recovery (Table 2).

Negative reported interactions were less common with only 8%^*^ (*n* = 6) of respondents indicating they experienced this. The most frequently reported concerns included: feeling triggered when offered substances while in hospital (*n* = 2), other patients intruding in their care (*n* = 2), and feeling unsafe or judged (*n* = 1).

### Access to cultural, spiritual, and psychosocial supportive services

Over half of respondents (*n* = 48, 55%) indicated that cultural and spiritual supports were important to their care. Among those who felt this way, 71% (*n* = 34) reported that their needs in this domain were met during their admission to the R2R unit (Table 3).

**Table 3:** Access to cultural, spiritual, and psychosocial supports and perceived barriers, May 2024 – April 2025 (*n* = 87)

Supportive Care	Total* n = 87 (%)
**Do you feel that cultural and spiritual supports are important to care?** ^**a**^	
Yes	48 (55)
No	12 (14)
Not sure	11 (13)
No opinion	11 (13)
** If you answered ‘yes’ to the above**,** were these needs met? (*n*** **= 48)**	
No	6 (13)
Yes	34 (71)
Not sure	5 (10)
No opinion	3 (6)
** If you answered ‘yes’ to the above**,** what contributed to these needs being met? (*n*** **= 34)**^b^	
Access to an Indigenous Wellness Liaison^c^	19 (83)
Was easily able to access cultural/religious services	18 (53)
Had access to cultural food	15 (44)
There were opportunities for ceremonial practices	14 (41)
Had good communication with others in cultural community	14 (41)
Was easy to communicate with spiritual Elders	12 (35)
Was able to access cultural medicines/treatment	11 (32)
Other^1^	2 (6)
** If you answered ‘no’ to the above**,** what contributed to these needs not being met? (*n*** **= 4)**	
There were barriers to accessing cultural/religious services	2 (50)
There was a lack of access to cultural food	2 (50)
There was a lack of opportunities for ceremonial practices	2 (50)
It was difficult to communicate with spiritual Elders	2 (50)
Was difficult to access cultural medicines/treatment	2 (50)
Was not able to access and Indigenous Wellness Liaison	1 (25)
There was a lack of communication with the cultural community	0 (0)
Other^2^	2 (50)
**Among those that identified as being Indigenous**,** did a visit by an Indigenous Wellness Liaison help to meet your cultural and spiritual needs?** (***n =*** **30)**^**d**^	
Completely	17 (57)
Mostly	1 (3)
Somewhat	7 (23)
Not at all	1 (3)
Not sure	4 (13)

* signifies over 10% missing data, the percentages reflect the proportion of total respondents to those questions

Imputations for incomplete responses presumed respondents did not check any items for these questions. Percentages reflect a denominator of 87 except in identified cases

a: Missing data: *n* = 5; b: missing data: *n* = 2; c: reflects a denominator of 23 Indigenous respondents; d: indicates percentages were calculated using a denominator of 33 respondents who were visited by and Indigenous Wellness Liaison

Other Responses: 1:Other responses included: ability to bring religious book, great experience overall; 2:Other responses included: denied smudging, barriers to access

Several factors were identified as contributing to the successful provision of culturally safe care. These included: access to an IWL support (*n* = 19, 83%*), the perception of ease in accessing cultural or religious services (*n* = 18, 53%), availability of traditional, cultural foods (*n* = 15, 44%), opportunities for ceremonial practices (*n* = 14, 41%), communication with cultural community members (*n* = 14, 41%), and communication with spiritual Elders (*n* = 12, 35%). Access to cultural medicines or treatments was reported to be available by 32% (*n* = 11). In contrast, 4 individuals reported their cultural and spiritual needs were not met, citing barriers to accessing culturally safe services, Indigenous programming, or providers of cultural and spiritual care (Table 3).

When asked specifically about visits from an IWL, 52%^*^ (*n* = 17) of Indigenous respondents who were visited by an IWL felt the visit completely met their cultural and spiritual needs. A smaller number of Indigenous respondents reported that these needs were mostly (*n* = 1, 3%*) or somewhat (*n* = 7, 21%*) met. Just one Indigenous respondent (3%) stated their needs were not met at all. Notably, 9%* (*n* = 3) of Indigenous respondents indicated they were not visited by an IWL (Table 3).

### Satisfaction with the quality of care provided and R2R’s physical environment

While admitted to R2R, most participants (*n* = 53, 61%) rated the quality of care as ‘excellent,’ while an additional 30% (*n* = 26) rated it as ‘good.’ Only a small proportion rated it as ‘okay’ (*n* = 6, 7%) or ‘poor’ (*n* = 2, 2%) (Table S1). Perceived quality of care did not differ significantly among youth. Specifically, when comparing participants less than 25 years of age to those 25 and older, there was no difference in the proportion of participants who ranked care as ‘excellent’ or good.’ Subgroup differences by gender were observed, such that 98% (*n =* 47) of men ranked care as ‘excellent’ or ‘good’ while only 88% (*n* = 29) of women and 88% (*n =* 7) of participants identifying in ‘other’ categories including non-binary, two-spirit, or transgender, ranked care as ‘excellent’ or ‘good’ (Table S2). Peer support services were reported to be helpful by 85% (*n* = 74) of respondents. Among this group, reasons cited included: feeling understood by peers (*n* = 59, 80%), connection with peers (*n* = 57, 77%), and having enough time to talk (*n* = 54, 73%). Among those who did not find peer support helpful (*n* = 12), the most common concern was not having enough time with peer support workers (*n* = 7, 58%) (Table S3).

Over three-quarters (*n* = 75, 86%) of participants reported the physical environment of R2R to be comfortable. Specific factors contributing to this included: patient rooms of adequate size (*n* = 64, 85%), well-lit spaces (*n* = 61, 81%), finding the environment culturally welcoming and safe (*n* = 60, 80%), and a sense of privacy (*n* = 61, 81%). Among those who felt uncomfortable in the physical environment (*n* = 10, 11%), reasons included: feeling the space was too much like a hospital (*n* = 6, 60%), a lack of common spaces (*n* = 6, 60%), and difficulties approaching the nursing station (*n* = 5, 50%) (Table S3). Comparatively, both Indigenous and non-Indigenous participants had roughly equal perceptions of the unit’s physical environment and the same reported reasons for not feeling comfortable (as described above).

Almost all respondents (*n* = 84, 97%) reported feeling physically safe on the unit, which was attributed to the securement of their belongings (*n* = 64, 76%), kind behavior from other patients (*n* = 55, 65%), and trust in staff protection (*n* = 59, 70%). Security personnel were also a comfort for many (*n* = 52, 62%). Only two respondents reported feeling unsafe, citing discomfort with security or feeling unprotected by staff (Table S3). Notably, 100% (*n* = 43) of Indigenous participants reported feeling safe on the unit. Additionally, 100% (*n =* 33) of women, 100% (*n =* 8) of those who identified their gender as something other than man or woman, and 100% (*n =* 11) of those aged less than 25 felt physically safe on the unit (Table S2).

Regarding patient perceptions of fairness and equity of treatment by healthcare staff, the majority (*n* = 71, 82%) did not feel they were treated unfairly due to their race or cultural background. However, 9% (*n* = 8) reported feeling unfairly treated. Among those, common concerns included: assumptions made about them, delays in services, and disrespectful tone (Table S3).

### Overall impressions of the R2R model of care

Participants highlighted key aspects of the R2R model that distinguished it from previous withdrawal management experiences. Nearly three-quarters (*n* = 52, 73%^*^) appreciated having privacy through single rooms. Over half reported feeling safer (*n* = 40, 56%^*^) and receiving better management of withdrawal symptoms (*n* = 39, 55%). Other frequently cited benefits included: having other medical needs addressed (*n* = 42, 59%), access to therapies not available elsewhere (*n* = 34, 48%), and improved discharge planning (*n* = 33, 46%) (Table S4). The majority (*n* = 77, 95%^*^) of participants reported they would recommend the program to friends or family if in need (Table S4). Notably, 100% (*n =* 7) of those who identified their gender as something other than man or woman and 100% (*n =* 10) of those aged less than 25 would recommend R2R (Table S2). Feedback collected from options for narrative responses within the survey highlighted the holistic, trauma-informed, and compassionate approach as key components. Comments emphasized the program’s ability to provide timely access to care, comprehensive aftercare planning, and a supportive, nonjudgmental environment. One participant wrote, *“This is the only place I could find intake immediately. You guys are saving lives.”* Another simply summarized their experience as, *“The best detox I ever went to—all my needs were met.”*

Regarding R2R’s primary goal, withdrawal management, 88%^*^ (*n* = 68) of participants reported adequate symptom relief during their admission. However, only 67% (*n =* 4) of those who identified their gender as something other than man or woman felt their withdrawal was adequately managed (Table S2). A total of 91%^*^ (*n* = 71) of participants felt that the R2R unit helped them to achieve their treatment goals, with pharmacologic therapy (*n* = 39, 54%^*^), and peer support (*n =* 33, 46%^*^) cited as the most helpful.

When asked whether participants had wanted to leave the R2R program early, one-third (*n* = 29, 33%) said ‘yes’. Among those who thought about leaving early (but decided to stay), the most common reason for wanting to leave was a lack of engaging activities offered (*n* = 12, 41%), followed by restrictive visiting or smoking rules (*n* = 5, 17% each), and unmanaged withdrawal symptoms (*n* = 4, 14%). Among the 62% (*n* = 54) of respondents who did not feel compelled to leave early, factors for this included: well-managed withdrawal symptoms (*n* = 43, 80%,), supportive staff (*n* = 42, 78%), and sufficient food (*n* = 30, 56%) (Table S4).

## Discussion

### Summary of Findings

Participants admitted to the R2R’s hospital-based withdrawal management unit overall reported positive experiences with the physical environment, staff interactions, and withdrawal management, and many felt the unit supported their treatment goals. These findings suggest that hospital-based withdrawal management models that integrate interdisciplinary, trauma-informed, and culturally safe care may improve patient experiences during acute withdrawal. However, gaps in psychosocial programming highlight important opportunities for improving engagement and retention during withdrawal management.

### Implications

Prior research has identified high rates of PID in withdrawal management settings, with common triggers including unmanaged symptoms and negative staff interactions [16, 25]. In contrast, this evaluation demonstrates the positive experiences that can be achieved when care is delivered through a culturally safe, trauma-informed, and comprehensive model with timely access to supports. While our findings support an integrated approach, one-third of participants still reported wanting to leave early, citing boredom and a lack of activities rather than clinical concerns. These findings highlight the importance of combining clinical care with recovery-oriented programming to maintain motivation and reduce disengagement.

One of the most distinctive features of the R2R model is its integration of Indigenous-specific programming, co-led by the IWR team. This stands in contrast to traditional withdrawal management settings, where cultural supports are minimal or absent. Research consistently shows that Indigenous people face elevated rates of PID and disengagement due to systemic racism, lack of cultural safety, and the absence of traditional healing practices [11, 26, 27]. In this evaluation, access to IWL services and traditional supports were associated with strong perceptions of cultural safety among Indigenous participants, reinforcing the importance of embedding Indigenous-led supports within withdrawal management programs. However, a small number of Indigenous respondents indicated that they were not visited by an IWL, and others cited difficulty accessing cultural supports, often linked to staffing limitations. These findings reinforce that Indigenous programming must be seen as essential, not supplementary, and that resourcing must match this commitment to ensure Indigenous programming is prioritized in care delivery.

Peer support was a unique and widely appreciated feature of the R2R model of care. Participants consistently emphasized the value of peer support within the care environment. This echoes findings in past literature, which highlight that the unique position of peer support workers grounded in shared lived experience enables them to offer empathy, relatability, and motivational support in acute-care settings [28]. Yet even this strength revealed system capacity challenges: participants who did not find peer support helpful often cited limited availability and insufficient time for connection. Peer support was not always available, potentially leaving gaps in care continuity. Similarly, occupational therapy and IWL services were only offered on weekdays, which may have contributed to inconsistent access. These findings indicate that while the R2R model includes innovative components, their impact may be constrained by staffing structures and scheduling.

In addition, difficulty with obtaining access to supports extended to psychosocial services (i.e., relapse prevention planning, group-based motivational enhancement therapy). Lack of participation could be due to various reasons, including staffing constraints, less scheduled programming on weekend days, and severity of withdrawal. Given the well-documented role of psychosocial care in improving retention and long-term recovery [29], this gap raises concerns. These findings emphasize the need for increased staffing and potentially extended training for psychosocial services to ensure more consistent access.

Additional exploratory analyses examining responses by gender and age revealed generally similar perceptions of care across groups, though slightly lower proportions of women and participants identifying their gender as other reported rating care as “excellent” or “good,” and those identifying as other gender were somewhat less likely to report adequate withdrawal management. While these findings should be interpreted cautiously due to small subgroup sizes, they highlight the importance of continued attention to how diverse patient populations experience withdrawal management services.

### Limitations

Limitations for this survey include its cross-sectional nature, small sample size, and single-site design. Given the timing of survey administration (i.e., close to discharge), selection bias may be present (as those individuals who may have prematurely discharged may not have had the opportunity to share their perspectives). Furthermore, participants may not have had the opportunity to share nuanced or complex feedback given the (predominantly) quantitative nature of the survey.

## Conclusion

This survey sought to understand experiences among individuals admitted to a new hospital-based withdrawal management unit that offered timely, culturally safe, trauma informed and comprehensive care. Overall, participants reported positive experiences with care delivery within the unit, suggesting that hospital-based withdrawal management can be delivered in a way that prioritizes patient safety, respect, and comfort. Many participants indicated that admission supported their individual treatment goals, highlighting the potential role of hospital-based withdrawal management as an entry point into broader addiction treatment and recovery pathways. These findings illustrate the benefit offering this model of care can have for individuals during acute withdrawal management and inform interventions that can further enhance clinical service delivery. Future research should focus on understanding the long-term impact of this model of care on treatment retention and recovery outcomes as well as strategies to address barriers identified in this evaluation such as limited access to structured psychosocial programming, peer support availability, and cultural services on weekends. Additionally, qualitative studies are needed to explore patient perspectives in greater depth, particularly among those who leave care early, to ensure future iterations of the program are even more responsive to patient needs. Scaling this model across other health systems may require tailored adaptations; however, the findings from this survey highlight that embedding trauma-informed, culturally safe, and interdisciplinary approaches within hospital-based withdrawal management can significantly improve patient experience and potentially reduce premature discharge. Continued investment in innovative, patient-centered care models like R2R will be critical in addressing the ongoing toxic drug crisis and improving outcomes for individuals with substance use disorders.

## Supplementary Information

Below is the link to the electronic supplementary material.

Supplementary Material 1

## Data Availability

The data that support the findings of this study are not openly available due to reasons of sensitivity and are available from the corresponding author upon reasonable request. Data are located in controlled access data storage at University of British Columbia. Formal request is direct through the Road to Recovery Indigenous data governance pathway.

## Abbreviations

R2R: Road to Recovery
IWR: Indigenous Wellness and Reconciliation
IWL: Indigenous Wellness Liaison
PID: Patient Initiated Discharge
SUD: Substance Use Disorder
SPH: St. Paul’s Hospital
HCAHPS: Hospital Consumer Assessment of Healthcare Providers and Systems
CMS: Centers for Medicare & Medicaid Services
