# Supplementary material for: Patient experiences and perceived efficacy of a newly implemented hospital-based withdrawal management unit in Vancouver, Canada: findings from the Road to Recovery evaluation
Source: Addict Sci Clin Pract. 2026 Apr 30;21:40. doi: 10.1186/s13722-026-00671-5 (PMC13135699; doi:10.1186/s13722-026-00671-5)
Supplement: Supplementary file 1 — Supplementary Material 1 [file 13722_2026_671_MOESM1_ESM.docx]

**SUPPLEMENTARY TABLES AND FIGURES**

**Figure S1**

***Local plant life traditionally used by Indigenous peoples incorporated into the unit environment***

**
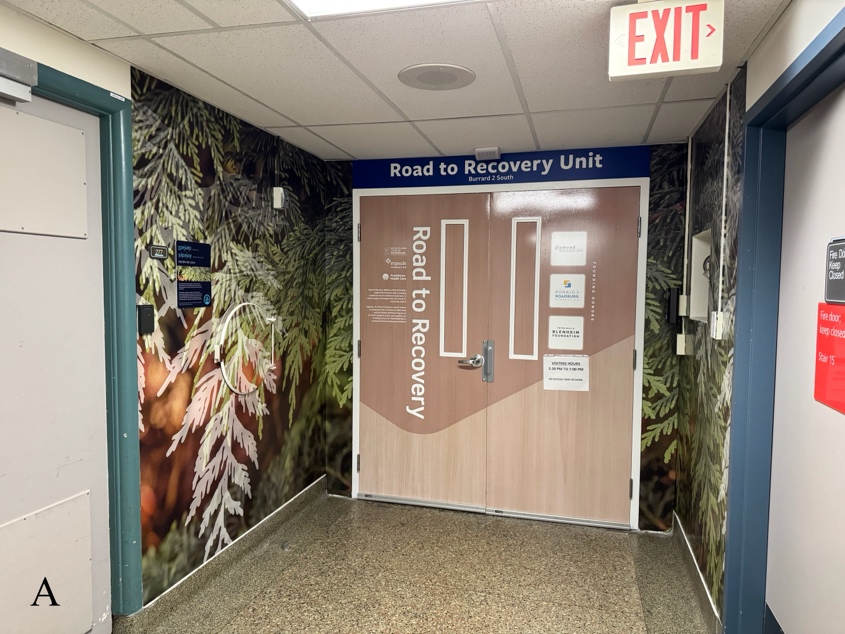

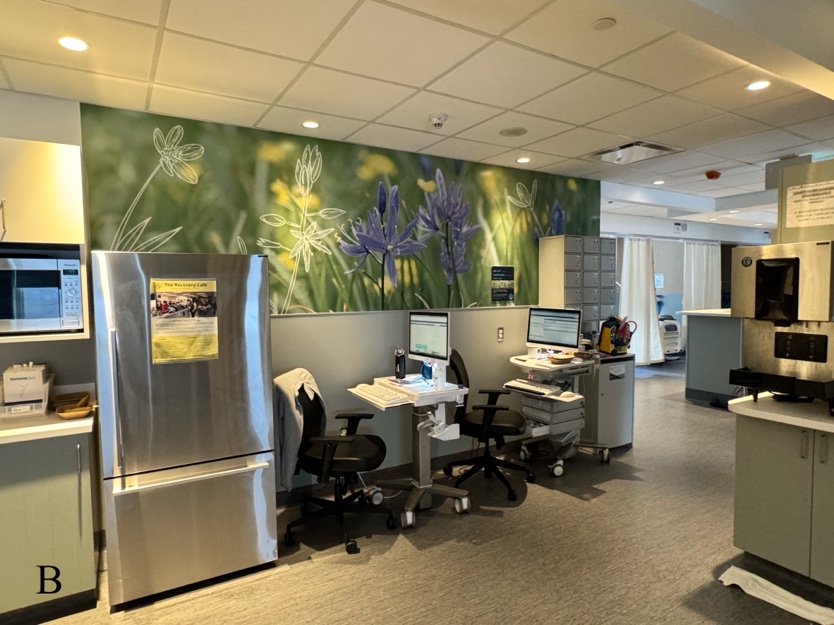
**

**Figure S1 Caption.** (A) Entrance to the acute unit depicting χpey̓əɬp / x̱ápay̓ay (western red cedar). Western red cedar is native to these lands and has traditionally been used by Indigenous peoples for spiritual cleansing and ceremony, as well as for building longhouses, canoes, house posts, regalia and clothing, bedding, and baskets. (B) Patient lounge area depicting spé-nxʷ / spánanexw (common camas). Common camas is native to these lands and has traditionally been used by Indigenous peoples for food and medicine.

**Figure S2**

*Most frequently used substances within the past 30 days (n=87)*

**Figure S2 Caption***:* Percentages reflect a denominator of 87. GHB: Gamma-hydroxybutyrate; Tranq-dope: unregulated fentanyl with tranquilizer contaminants, often xylazine; Benzo-dope: fentanyl with one or more benzodiazepine contaminants. Other responses included: ketamine, ecstasy, gabapentin, and Gravol

**Table S1**

*Services perceived to be most helpful in achieving treatment goals, May 2024 - 2025 (n=72)*

| **Service(s) most helpful in achieving treatment goals (*n*=72)** | ***n* (%)** |
| --- | --- |
| Pharmacologic therapy | 39 (54) |
| Access to an Indigenous Wellness Liaison* | 23 (53) |
| Residential treatment | 30 (42) |
| Detox | 29 (40) |
| Community addiction provider | 23 (32) |
| Transitional care beds | 21 (29) |
| Primary care provider | 20 (28) |
| Recovery housing | 20 (28) |
| Psychosocial services | 18 (25) |
| Housing support services | 16 (22) |
| Connection to subspecialty care | 11 (15) |
| Harm reduction supplies | 11 (15) |
| Harm education | 11 (15) |
| Community health center | 10 (14) |
| Daytox | 7 (10) |
| Services for the criminal justice system | 3 (4) |
| Employment/vocational services | 3 (4) |
| **Table S1 Caption:** Imputations for incomplete responses presumed respondents did not check any items for these questions. Percentages reflect a denominator of 72 excluding the category ‘Access to an Indigenous Wellness Liaison’ for which the total denominator of 43 was used to reflect the number of survey respondents who identified as Indigenous.  **Table S2**  *Satisfaction and perceptions of care divided by gender and age groups, May 2024 – 2025 (*n*=87)*   \|  \| **Men n (%)** \| **Women n (%)** \| **Other n (%)** \| **Age < 25 n (%)** \| **Age ≥ 25 n (%)** \| \| --- \| --- \| --- \| --- \| --- \| --- \| \| **How would you describe the quality of clinical care you received while admitted to SPH's R2R unit?** \| \| \| \| \| \| \| Excellent or good \| 47 (98) \| 29 (88) \| 7 (88) \| 11 (92) \| 65 (92) \| \| Okay or poor \| 1 (2) \| 4 (12) \| 1 (12) \| 1 (8) \| 6 (8) \| \| **Did you feel your withdrawal was adequately managed?*** \| \| \| \| \| \| \| Yes \| 37 (88) \| 29 (91) \| 4 (67) \| 9 (90) \| 56 (88) \| \| No \| 5 (12) \| 3 (9) \| 2 (33) \| 1 (10) \| 8 (12) \| \| **While admitted to the R2R unit, did you feel physically safe on the unit** \| \| \| \| \| \| \| Yes \| 47 (96) \| 33 (100) \| 8 (100) \| 11 (100) \| 69 (97) \| \| No \| 2 (4) \| 0 (0) \| 0 (0) \| 0 (0) \| 2 (3) \| \| **Would you recommend R2R to family or friends in need?*** \| \| \| \| \| \| \| Yes \| 40 (95) \| 30 (97) \| 7 (100) \| 10 (100) \| 59 (94) \| \| No \| 2 (5) \| 1 (3) \| 0 (0) \| 0 (0) \| 4 (6) \| \| **Table S2 Caption**: ** signifies over 10% missing data, the percentages reflect the proportion of total respondents within each demographic subgroup.* Participants identifying as “other” gender included non-binary, two-spirit, and transgender; results should be interpreted with caution due to small sample size. \| \| \| \| \| \| | |

**Table S3**

*Perceived Quality of Care, Environment, and Overall Impressions of the Road to Recovery (R2R) (n=87)*

| **Perceived Quality of Care and Environment** | **Total***  **n=87 (%)** |
| --- | --- |
| **If you found peer support helpful, what contributed to this? (*n*=74)**^a^ | ***n* (%)** |
| Could understand me/my experiences | 59 (80) |
| Could connect with them | 57 (77) |
| Had enough time to talk with them | 54 (73) |
| They were focused on advocating for me | 47 (64) |
| They were available when I needed them | 45 (61) |
| They supported me in reaching my goals | 44 (59) |
| They helped motivate me | 44 (59) |
| Conversations with them motivated me to stay | 31 (42) |
| Other^1^ | 8 (11) |
| **If you found peer support unhelpful, what contributed to this? (*n*=12)** | ***n* (%)** |
| Did not have enough time with them | 7 (58) |
| I could not connect with them | 3 (25) |
| They were unavailable | 2 (17) |
| They were more focused on helping the medical team than advocating for me | 2 (17) |
| Felt they did not understand me | 1 (8) |
| They were unsupportive | 0 (0) |
| Other^2^ | 2 (17) |
| **Did you find that the physical environment was comfortable?**^b^ | ***n* (%)** |
| Yes | 75 (86) |
| No | 10 (11) |
| **If answered ‘yes’ above, what contributed to a comfortable physical environment? (*n*=75)**^b^ | ***n* (%)** |
| Patient rooms were a good size | 64 (85) |
| Felt comfortable approaching the nursing station | 62 (83) |
| There was adequate privacy | 61 (81) |
| Culturally welcoming and safe | 60 (80) |
| It was a well-lit space | 61 (81) |
| Having a phone was helpful | 47 (63) |
| Nursing station was not too loud | 44 (59) |
| Presence of security made me feel safe | 45 (60) |
| Presence of security was not noticeable | 37 (49) |
| Enough common spaces to connect with others | 33 (44) |
| Did not feel like a hospital | 32 (43) |
| Other^3^ | 7 (9) |
| **If answered ‘non’ above, what contributed to an uncomfortable physical environment? (*n*=10)** | ***n* (%)** |
| Felt too much like a hospital | 6 (60) |
| Not enough common spaces | 6 (60) |
| Approaching the nursing station was intimidating | 5 (50) |
| Not enough privacy | 4 (40) |
| Nursing station was too loud | 4 (40) |
| Not culturally welcoming or safe | 2 (20) |
| Presence of security was noticeable | 2 (20) |
| Patient rooms were too small | 1 (10) |
| Security did not make me feel safe | 1 (10) |
| Not a well-lit space | 0 (0) |
| Having a phone was triggering | 0 (0) |
| Other^4^ | 2 (20) |
| **Did you feel physically safe on the unit?**^b^ | ***n* (%)** |
| Yes | 84 (97) |
| No | 2 (2) |
| **If answered ‘yes’ above, what contributed to feeling physically safe? (*n*=84)**^a^ | ***n* (%)** |
| Felt my belongings were secure | 64 (76) |
| Felt like staff could protect me | 59 (70) |
| The unit felt protected from the public | 59 (70) |
| Security guards made me comfortable | 52 (62) |
| Other patients were kind | 55 (65) |
| Other^5^ | 7 (8) |
| **If answered ‘no’ above, what contributed to not feeling physically safe? (*n*=2)** | ***n* (%)** |
| Security guards made me uncomfortable | 1 (50) |
| Felt like staff could not protect me | 1 (50) |
| Other patients were verbally/physically aggressive | 0 (0) |
| Was scared belongings would be stolen | 0 (0) |
| The unit felt too accessible to the public | 0 (0) |
| Other | 0 (0) |
| **Did you feel you received unfair treatment due to race or cultural background?**^b^ | ***n* (%)** |
| Yes | 8 (9) |
| No | 71 (82) |
| Not sure | 1 (1) |
| No opinion | 5 (6) |
| **If answered ‘yes’ above, what contributed to perceptions of unfair treatment? (*n*=5)** | ***n* (%)** |
| Assumptions were made about me or a worker held stereotypes | 4 (80) |
| Services were delayed | 3 (60) |
| Was talked down to or a rude tone was used | 2 (40) |
| Was denied services | 2 (40) |
| The doctor/nurse acted like they were better than me | 2 (40) |
| Healthcare worker was not respectful of cultural practices/traditions/ceremonies | 1 (20) |
| Healthcare worker did not take me seriously | 1 (20) |
| The doctor/nurse acted like I was not smart | 1 (20) |
| Care was physically rough | 0 (0) |
| Was discharged too early | 0 (0) |
| Received poorer services than others | 0 (0) |
| The doctor/nurse acted afraid of me | 0 (0) |
| Other^6^ | 1 (20) |
| **Table S3 Caption:** *** signifies over 10% missing data, the percentages reflect the proportion of total respondents to those questions  Imputations for incomplete responses presumed respondents did not check any items for these questions. Percentages reflect a denominator of 87 except in identified cases  a:Missing data: n=1; b: missing data: n=2  Other Responses: 1:Other responses included: they went the extra mile, friendly and considerate, kind, made me feel comfortable and safe, felt respected, eased my discomfort; 2:Other responses included: interruptions prevented connection, did not speak to one; 3:Other responses included: Able to keep personal items, having a tablet was helpful, allowed comfort items like blankets, access to snacks, allowed independence; 4:Other responses included: no common area, few opportunities to smoke; 5:Other responses included: self-defence skills, medical needs being met, doors were locked, people are screened when entering the unit, felt safe from myself; 6:Other responses included: smudging was denied | |
|  | |

**Table S4**

*Overall impressions: Factors Influencing Early Departure, Retention, Comparative Detox Experiences, and Program Satisfaction (*n*=87)*

| **Overall Impressions** | **Total***  **n=87 (%)** |
| --- | --- |
| **Did you want to leave the Road to Recovery early?**^a^ | ***n* (%)** |
| Yes | 29 (33) |
| No | 54 (62) |
| **If answered ‘yes’ above, what contributed to wanting to leave early? (*n*=29)**^b^ | ***n* (%)** |
| Not enough activities | 12 (41) |
| Restrictive smoking rules | 5 (17) |
| Visiting hours too restrictive | 5 (17) |
| Too little food | 4 (14) |
| Other patient behaviours made me want to use | 4 (14) |
| Withdrawal symptoms not managed well | 4 (14) |
| I was not ready to make a change | 4 (14) |
| Staff made me feel unwelcome | 4 (14) |
| Poor quality food | 3 (10) |
| Having a phone was triggering | 1 (3) |
| Physical environment made me uncomfortable | 0 (0) |
| Did not feel culturally safe | 0 (0) |
| Other^1^ | 12 (41) |
| **If answered ‘no’ above, what contributed to wanting to stay? (*n*=54)**^a^ | ***n* (%)** |
| My withdrawal symptoms were managed well | 43 (80) |
| Staff made me feel comfortable | 42 (78) |
| Enough food was provided | 30 (56) |
| Other patients made me feel comfortable | 26 (48) |
| Physical environment made me feel comfortable | 25 (46) |
| Food was good quality | 24 (44) |
| I had access to cultural and spiritual supports | 19 (35) |
| Visiting hours were good | 15 (28) |
| Enough smoking time was allotted | 13 (24) |
| Lots of activities to keep me busy | 5 (9) |
| Other^2^ | 6 (11) |
| **How did experiences at Road to Recovery differ from previous detox experiences? (*n*=71)** | ***n* (%)** |
| I had more privacy with a single room | 52 (73) |
| My other medical needs were evaluated and addressed | 42 (59) |
| Felt safer on this unit | 40 (56) |
| Withdrawal symptoms were better managed | 39 (55) |
| I was provided therapies not available at other detox centers | 34 (48) |
| I did not have to wait long to receive services | 34 (48) |
| The plan for my care after detox was more organized | 33 (46) |
| I was connected to specialist services | 29 (41) |
| Same day admission to a withdrawal management center | 27 (38) |
| I had more time with the physician | 25 (35) |
| I had access to an IWL | 25 (35) |
| There were more activities | 11 (15) |
| Certain activities were more available | 9 (13) |
| Other^3^ | 10 (14) |
| **Would recommend R2R to family or friends in need? (*n*=77)** | ***n* (%)** |
| Yes | 73 (95) |
| No | 4 (5) |
| **Table S4 Caption:** *** signifies over 10% missing data, the percentages reflect the proportion of total respondents to those questions  Imputations for incomplete responses presumed respondents did not check any items for these questions. Percentages reflect a denominator of 87 except in identified cases  a:Missing data: n=4; b:missing data: n=1  1:Other responses included: felt unworthy of care, had personal life problems or responsibilities outside of hospital, was too sick to be close to other patients, overheard staff say unkind things, unable to exercise, pain management was inadequate, missed being home, stay was too long, felt like giving up; 2:Other responses included: friendly staff, was reminded of my goals, able to receive cultural food, detoxed at a good rate; 3:Other responses included: felt genuine care and empathy, less strict than other detoxes, received prenatal care, allowed personal items | |
